# Differential expression of miRNAs revealed by small RNA sequencing in traumatic tracheal stenosis

**DOI:** 10.3389/fgene.2023.1291488

**Published:** 2024-01-08

**Authors:** Wentao Li, Jinmei Wei, Pingping Huang, Yuhui Wei, Li Chang, Guangnan Liu

**Affiliations:** ^1^ Department of Respiratory and Critical Care Medicine, The Second Affiliated Hospital of Guangxi Medical University, Nanning, China; ^2^ Guangxi Medical University, Nanning, China; ^3^ Department of Dermatology of Shenzhen People’s Hospital, The Second Clinical Medical College of Jinan Uninversity, The First Affiliated Hospital of Southern University of Science and Technology, Shenzhen, China

**Keywords:** traumatic tracheal stenosis, miRNAs, small RNA sequencing, differential expression, Gene Ontology and Kyoto Encyclopedia of Genes and Genomes enrichment analysis

## Abstract

**Introduction:** Traumatic tracheal stenosis (TTS) is a major cause of complex difficult airways, without clinically definitive efficacious drugs available. The aim of this study was to provide a general view of interactions between micro and messenger ribonucleic acids (miRNAs and mRNAs) and many potential mechanisms in TTS *via* small RNA sequencing.

**Methods:** In this study, the identification of miRNAs was completed using small RNA sequencing and samples from four TTS patients and four normal control cases. By using bioinformatics tools, such as miRanda and RNAhybrid, for identifying the candidate target genes of miRNAs with differential expression in each sample, Gene Ontology and Kyoto Encyclopedia of Genes and Genomes were employed for enriching the predicted target genes of miRNAs with differential expression based on the correspondence between miRNAs and their target genes. We detected the expression of the candidate miRNAs using quantitative real-time polymerase chain reaction (qRT-PCR).

**Results:** Twenty-four miRNAs with significant differential expression were identified, including 13 upregulated and 11 downregulated ones. Bioinformation technology was adopted to predict 2,496 target genes. These miRNA-target genes were shown to be primarily enriched in cells and organelles with catalytic activity and binding function, such as binding proteins, small molecules, and nucleotides. Finally, they were observed to process into TTS through the intercellular and signal regulation of related inflammatory signaling and fibrosis signaling pathways. QRT-PCR confirmed the upregulation of miR21-5p and miR214-3p and the downregulation of miR141-3p and miR29b-3p, which was expected to become a high-specific miRNA for TTS.

**Conclusion:** Among all the miRNAs detected, 24 miRNAs demonstrated differential expression between the TTS and normal control groups. A total of 2,496 target genes were predicted by bioinformation technology and enriched in inflammatory and fibrotic signaling pathways. These results provide new ideas for further studies and the selection of targets for TTS in the future.

## Introduction

Traumatic tracheal stenosis (TTS) is mostly caused by tracheal intubation and tracheostomy. Its increased morbidity is associated with the development of mechanical-assisted ventilation (Murgu et al., 2016; Ghiani et al., 2022). At present, the treatment of TTS patients has no specific drug and mainly relies on bronchoscope interventional therapy and surgical treatment (Freitag et al., 2007). However, interventional therapy and surgical treatment can cause physical and psychological damage to TTS patients (THORNTON et al., 2006). Therefore, inhibiting and reversing the formation of tracheal granulation tissues are crucial areas of current research. Moreover, the molecular pathogenesis underlying the occurrence and development of TTS remains elusive.

Micro ribonucleic acids (miRNAs) as small non-coding RNAs inhibit or degrade messenger RNAs (mRNAs) by binding to the 3′-untranslated regions (3′-UTRs) of target mRNAs. They widely regulate life processes, including cell growth, proliferation, apoptosis, differentiation, and body metabolism (Rupaimoole and Slack, 2017; Pu et al., 2019). Inhibiting the expression of miR-1275 has been reported to possibly alleviate airway stenosis (ZHANG et al., 2021). Because of less research on miRNAs in TTS, this study aimed to investigate the influencing mechanism of miRNAs in the occurrence and development of TTS.

Small RNA sequencing high-throughput technology was used as the entry point to sequence TTS genes. In addition, the differential expression profile of miRNAs was drawn, and an miRNA–mRNA regulatory network was constructed in TTS. The purpose was to comprehensively reveal the biological functions of miRNAs in fibroblasts from multiple levels and dimensions. Thus, this provides a theoretical basis and precise molecular targets for the regulatory network of the pathogenesis of TTS.

## Materials and methods

### Patients

TTS patients were recruited upon the first diagnosis at the Department of Respiratory and Critical Care Medicine in The Second Affiliated Hospital of Guangxi Medical University (GXMU) from January 2019 to December 2020. All TTS patients were aged over 18 years, in whom TTS was caused by tracheal intubation or tracheotomy, and the patients were diagnosed by medical history, computed tomography (CT), pathology, and bronchoscopy. Patients with tracheal stenosis caused by other etiologies were excluded, including those with malignant and benign tumors and tuberculosis. TTS granulations were performed through bronchoscopy.

Patients in the normal control group were recruited from those who were over 18 years and those who have undergone a pulmonary lobectomy at the Department of Thoracic and Cardiovascular Surgery in The Second Affiliated Hospital of GXMU from January 2019 to December 2020. Normal bronchial mucosa was taken from the resected bronchus and confirmed by pathological analysis, which indicated no tumor infiltration, fibrosis, or inflammation.

The aforementioned samples were collected into an RNA preservation solution at 4°C overnight and stored at −80°C for subsequent experiments. The consent of the patients or their families was obtained before the collection of samples. All procedures gained the approval of the Ethics Association of the Second Affiliated Hospital of GXMU, with protocol reference no. 2019-KY(0109). These procedures were performed as per institutional guidelines.

### RNA extraction, quantification, and qualification

Tissues were ground thoroughly using a grinder after being melted at room temperature, followed by the extraction of total RNAs by TRIzol reagent (Takara, China), following the protocol of the manufacturer. RNAs were not degraded and contaminated, which was confirmed by 1% agarose gels. The NanoPhotometerVR spectrophotometer [IMPLEN, the United States of America (United States)] was utilized for testing the purity of RNAs. Then, a QubitVR RNA Assay Kit in QubitVR 2.0 Flurometer (Life Technologies, United States) was used for measuring the concentration of RNAs. The RNA Nano 6000 Assay Kit of the Agilent Bioanalyzer 2,100 system (Agilent Technologies, United States) was applied to assess the integrity of RNAs within a RIN value ranging between 6.0 and 8.0. The above RNAs were utilized for the sequencing of small RNAs and quantitative real-time polymerase chain reaction (qRT-PCR) analysis.

### Small RNA sequencing and data analysis

#### Small RNA library construction

A total of 3 μg total RNAs per sample were used for the small RNA library construction which were created by the use of the NEBNext Multiplex Small RNA Library Prep Set for Illumina (NEB, United States) following manufacturer’s instructions. The NEB 3′junction was accurately linked to the 3′end of the miRNA, siRNA, or piRNA. The reverse transcription primer was bound to the redundant 3′junction for end closure. The single-stranded deoxyribonucleic acid (DNA) adaptor was transformed into a double-stranded DNA fragment. The NEB 5′junction is bound precisely to the 5′end of the miRNA. The synthesis of the first-strand complementary DNA (cDNA) was completed via RNA reverse transcriptase. The double-strand cDNA synthesis was synthesized after the Illumina sequencing junction was ligated to the cDNA by PCR. Then, 8% polyacrylamide gel (the recovery of 140–160 bp target bands) was used to purify the PCR product and thus obtain cDNA libraries.

#### Small RNA sequencing

Following library construction, Qubit^®^ 2.0 was used for preliminary quantitative analysis. The insert size of the library was measured by Agilent 2,100 after it was diluted to 1 ng/L to ensure the acceptability of the library test. After this, pooling was carried out as per effective concentration and the target volume of downstream data. HiSeq 2,500/2000 was used for single-end 50 bp sequencing according to Illumia requirements. The workflow shown in the figure was followed when a bioinformatics analysis was conducted on the small RNA sequencing dataset. The raw datasets of sRNA were submitted to the Sequence Read Archive of the NCBI database.

#### Data analysis of miRNAs

Reads containing ploy N, with 5′ adapter contaminants, without 3’ adapter or the insert tag, containing ploy A or T or G or C and low-quality reads from raw data were removed. Q20, Q30, and GC-content of the raw data were calculated. The small RNA alignments were mapped to the reference sequence by Bowtie (Langmead et al., 2009) without mismatch to analyzing their expression on the reference. Mapped miRNA tags were used to look for known miRNA, and miRBase20.0 was used as the reference and modified software (Friedländer et al., 2012). Readcount values from the expression level analysis of miRNAs, which measured the differential expression of miRNAs, were used. Statistical analysis was used based on negative binomial distribution DESeq2 R package (Pu et al., 2019). The threshold Padj0.05, |log2 (foldchange)|>1, was set. In addition, significantly differentially expressed miRNAs were screened. The results were displayed as a volcano plot and a hierarchical clustering plot, respectively, following biological repeat characteristics.

#### Target gene prediction and Gene Ontology/Kyoto Encyclopedia of Genes and Genomes enrichment analysis

**FIGURE 1:**
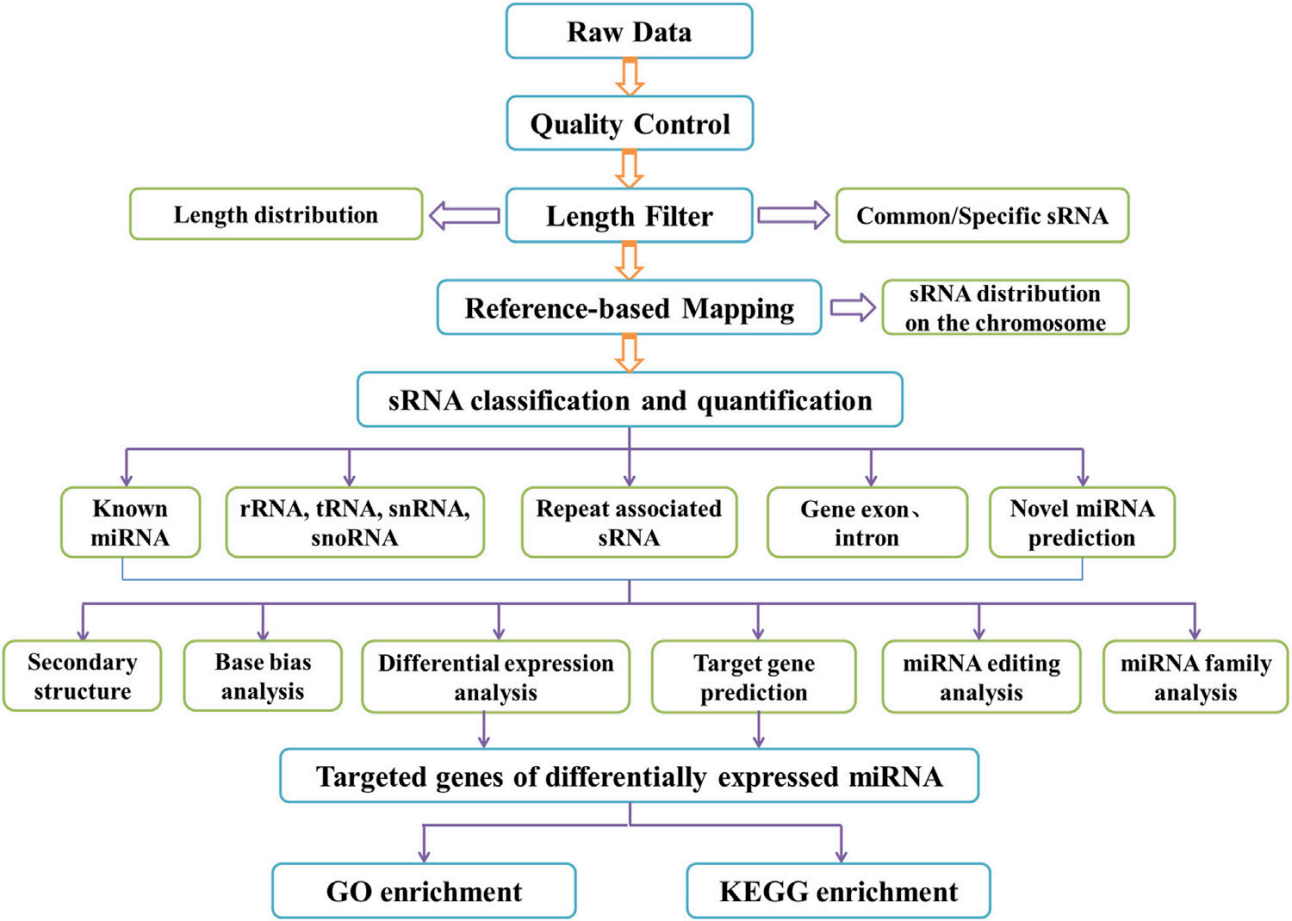
Flow diagram of bioinformatics analysis.

Bioinformatics tools miRanda and RNAhybrid were used for identifying the candidate target genes of miRNAs with differential expression in each sample, Gene Ontology (GO) and Kyoto Encyclopedia of Genes and Genomes (KEGG) were adopted for enriching the predicted target genes of miRNAs with differential expression based on the correspondence between miRNAs and their target genes (Kanehisa et al., 2008; Young et al., 2010). KOBAS software was used to test the statistically enrichment of the target genes candidates in the KEGG pathways (Mao et al., 2005). Figure 1 shows the flow diagram of the bioinformatics analysis.

#### Differential expression of miRNAs by qRT-PCR

Total RNAs were extracted from TTS and normal control tissues. In addition, an miRNA RT kit (Takara, China) was used for the RT of total RNAs into cDNAs. Next, qRT-PCR was carried out using the QuantStudioTM 5 Real-Time PCR System (Agilent Technologies) by use of SYBR Green PCR Kit (Takara), following the protocol of the manufacturer. Sangon Biotech (Shanghai, China) was utilized for the chemical synthesis of primers used for qRT-PCR. Table 1 shows the sequences of these primers. U6 was used as the internal reference, and 2^-△△CT^ was used as the relative expression level.

**TABLE 1:** Primers’ sequence for qRT-PCR.

Gene	Primer sequence
U6-Forward	CTCGCTTCGGCAGCACA
U6-Reverse	AAC​GCT​TCA​CGA​ATT​TGC​GT
miR-21-5p-Forward	CGG​CTT​ATC​AGA​CTG​ATG​TTG​AA
miR-214-3p-Forward	TTA​CAG​CAG​GCA​CAG​ACA​GGC
miR-141-3p-Forward	GCG​CTA​ACA​CTG​TCT​GGT​AAA​GAT​GG
miR-29b-3p-Forward	GCG​CTA​GCA​CCA​TTT​GAA​ATC​AGT​GTT

#### Statistical analysis

The statistical processing of the data and the creation of graphs were completed using *SPSS 26.0* and GraphPad Prism 8, respectively. All data were tested for normality, the *Student’s* t*-test* was applied to the data that fit the normal distribution, and the Mann–Whitney *U* test was applied to the data that did not fit the normal distribution. Statistical significance was defined to be *p* < 0.05.

## Results

### Summary of sequencing data

Four TTS (TS_1, TS_2, TS_3, and TS_4) and four normal control patients (Control_1, Control_2, Control_3, and Control_4) were recruited in this study for small RNA sequencing. Their general information is shown in Table 2. Illumina was used to construct and sequence eight small RNA libraries (including four TTS and four normal controls). In addition, 1,115 known miRNA matures and 984 miRNA precursors were identified in the samples of TTS and control groups. The summary of sequencing data is shown in Table 3. Moreover, 510, 509, 661, 446, 848, 886, 390, and 456 miRNA matures and 458, 459, 618, 403, 753, 765, 363, and 403 miRNA precursors were obtained from Control_1, Control_2, Control_3, Control_4, TS_1, TS_2, TS_3, and TS_4 libraries, respectively. Furthermore, 49 new matures and 52 new precursors were found in the eight libraries.

**TABLE 2:** General information of patients.

Patient	Sex	Age (years)	Diagnose
TS_1	Male	23	Tracheal stenosis after intubation
TS_2	Male	65	Tracheal stenosis after intubation
TS_3	Female	42	Tracheal stenosis after intubation
TS_4	Female	36	Tracheal stenosis after intubation
Control_1	Male	58	Lung squamous cell carcinoma
Control_2	Male	49	Lung squamous cell carcinoma
Control_3	Female	56	Lung adenocarcinoma
Control_4	Female	71	Lung adenocarcinoma

**TABLE 3:** Summary of sequencing data.

Type	Sum	Control_1	Control_2	Control_3	Control_4	TS_1	TS_2	TS_3	TS_4
Mature	1,115	510	509	661	446	848	886	390	456
Precursor	984	458	459	618	403	753	765	363	403
New mature	49	7	12	29	3	14	11	2	4
New precursor	52	7	12	31	3	16	12	2	4

**FIGURE 2:**
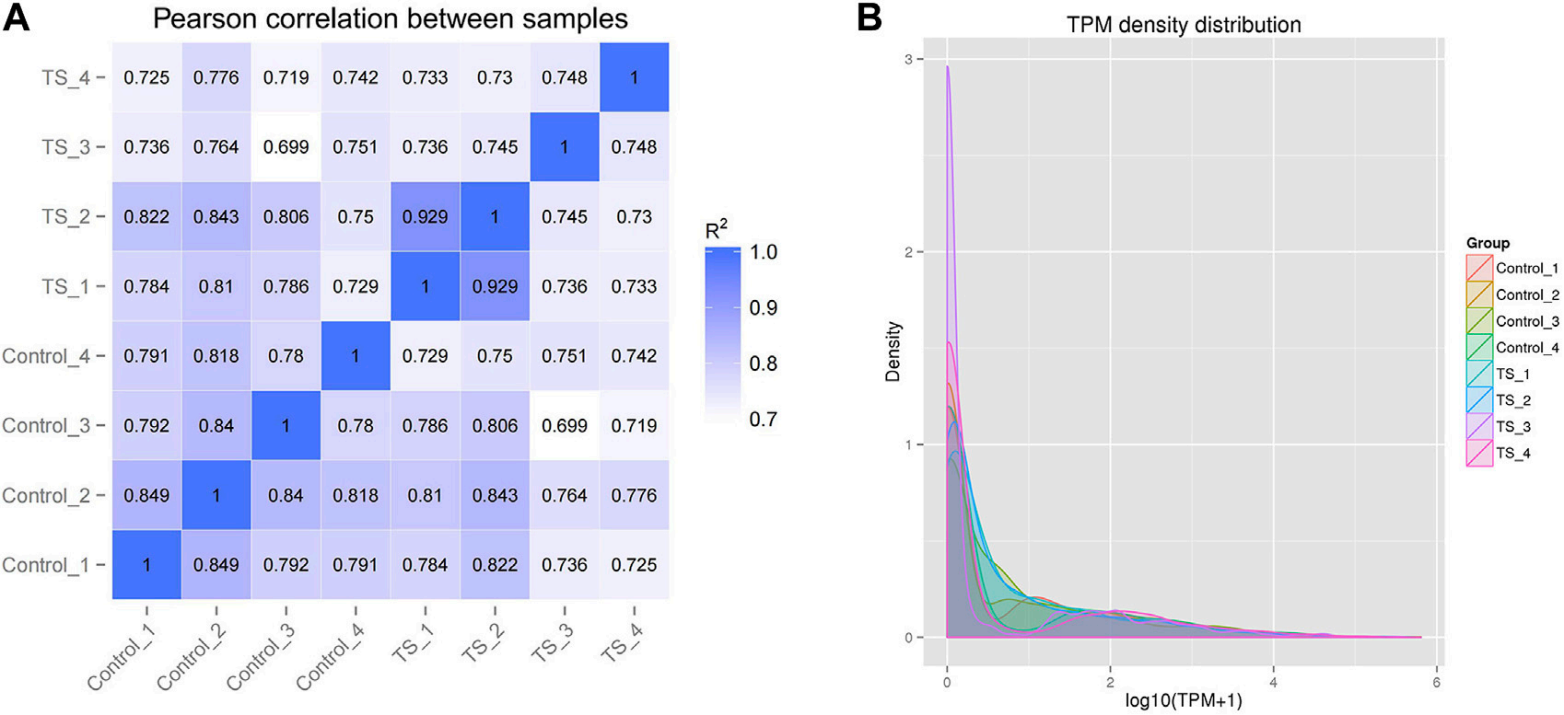
Correlation and TPM density distribution of miRNA expression in all samples. **(A)** Correlation between the samples of TTS and control groups; the abscissa and ordinate are the log10 (TPM +1) value of the sample, respectively. **(B)** TPM density distribution of miRNA expression in all samples; the abscissa and ordinate are the log10 (TPM +1) value of the miRNA and the density of the corresponding log10 (TPM +1), respectively. *R* ^2^: the square of the Pearson correlation coefficient.

The intra-group correlation coefficients for the control and TTS groups fluctuated between 0.78–0.849 and 0.729–0.99, respectively, whereas the inter-group ones for the two groups fluctuated between 0.699 and 0.843. This indicated the high similarity of expression patterns between the samples and good biological comparability and reproducibility (Figure 2A). According to the transcripts per million (TPM) density distribution of miRNA expression in each sample of tracheal stenosis and control groups, each sample showed basically the same overall gene expression. This indicates that the expression of miRNAs between samples was well assessed in terms of quality after TPM normalization, which was consistent with experimental requirements (Figure 2B).

### MiRNA profiling analysis

**FIGURE 3:**
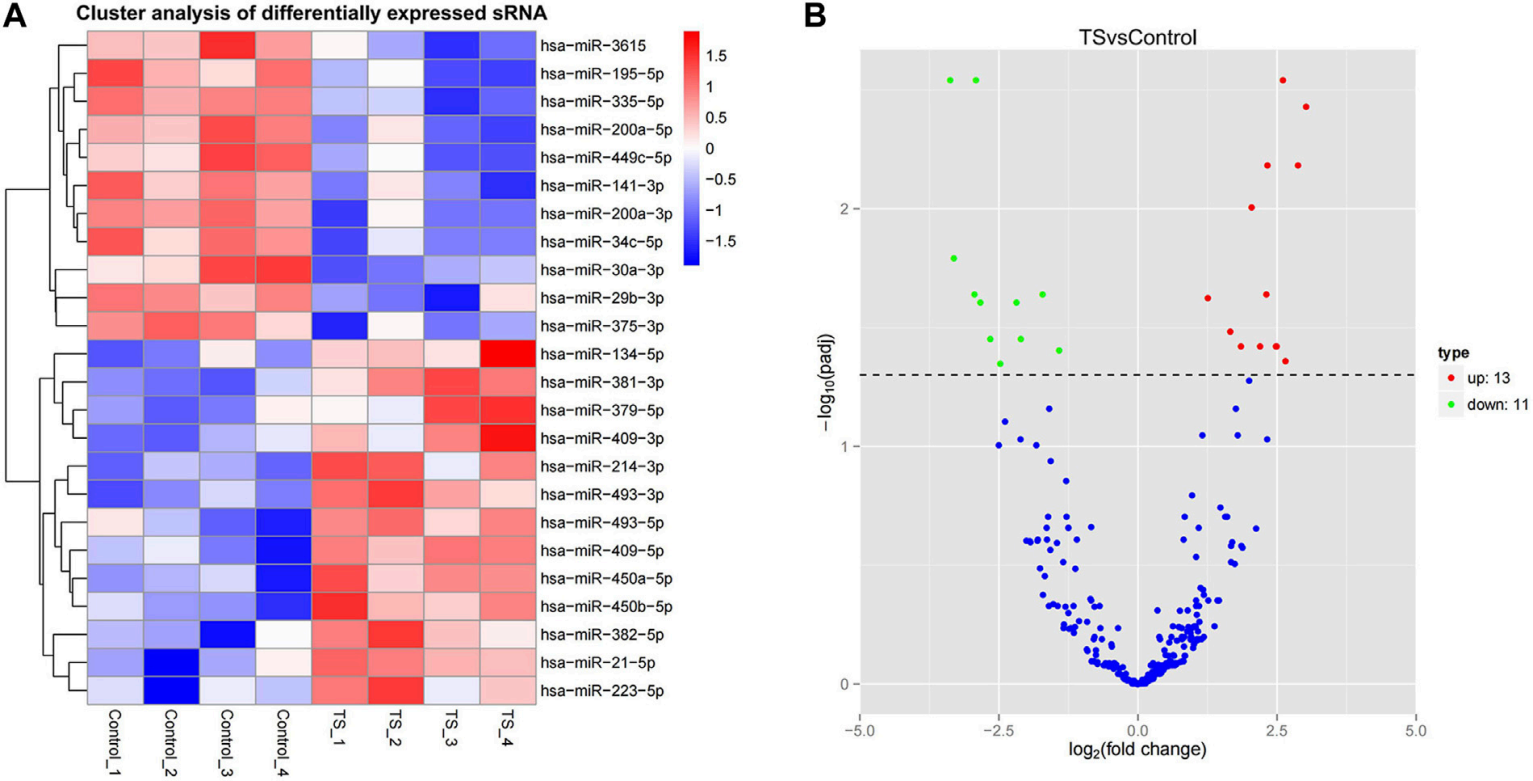
Differential miRNA expression profile. **(A)** Hierarchical clustering map, clustering with log10 (TPM +1) values; red and blue indicate highly and lowly expressed miRNAs, respectively. **(B)** Volcanic map; the abscissa in the volcano plot is the fold change of the miRNA expression in both groups (expressed as log2 fold change), and the ordinate is the significance level of expression difference (expressed as -log10 (padj)). Upregulated miRNAs are indicated by red dots, downregulated miRNAs are indicated by green dots, and miRNAs with no significant change are indicated by blue dots.

In this study, miRNAs from the TTS and normal control groups were further evaluated for significant differential expression by using |log2 fold change| > 1 and corrected *p*-value <0.05 as the cutoff. In addition, 24 miRNAs with significant differential expression were obtained. As shown in Supplementary Table S1 and Figure 3A, 13 were upregulated, namely, miR-450a-5p, miR-450b-5p, miR493-3p, miR-93-5p, miR-409-5p, miR-409-3p, miR-21-5p, miR-214-3p, miR-223-5p, miR-379-5p, miR-134-5p, miR-382-5p, and miR-381-3p. Meanwhile, 11 were downregulated, namely, miR-29b-3p, miR-30a-3p, miR-195-5p, miR-200a-3p, miR-200a-5p, miR-141-3p, miR-3615, miR-34c-5p, miR-375-3p, miR-449c-5p, and miR-335-5p. Hierarchical clustering and volcanic maps are shown in Figure 3.

### Target gene prediction and enrichment analysis

**FIGURE 4:**
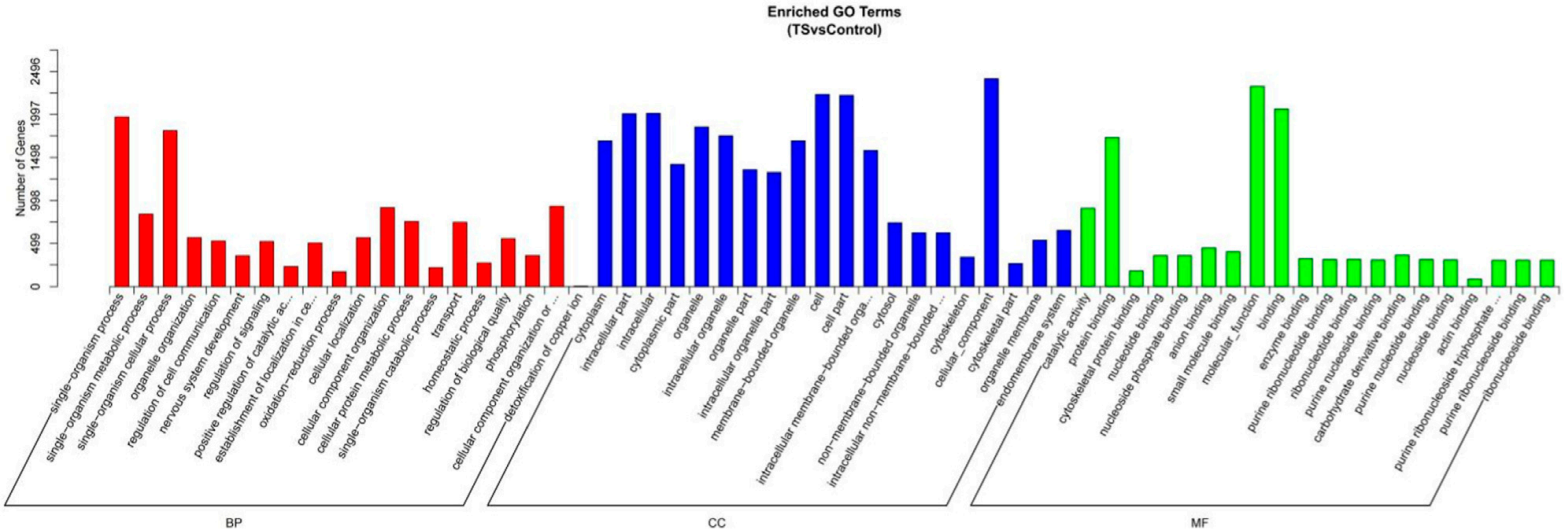
GO gene function classification of the target genes of miRNAs with differential expression. BP, biological processes; CC, cellular components; and MF, molecular functions.

The miRanda and RNAhybrid software were used to obtain 2,496 target genes through target gene prediction. Candidate gene sets were analyzed using GO and KEGG enrichment analyses (Supplementary Table S2). The GO enrichment analysis results are shown in Figure 4 and Supplementary Tables S3–S5. First, biological processes (BPs) are mainly enriched in “single-organism processes (gene number 1974),” “single-organism metabolic processes (gene number 907),” “single-organism cellular processes (gene number 1823),” “intercellular interaction regulation (gene number 528),” and “signal regulation (gene number 528).” Cellular components (CCs) were mainly enriched in “cytoplasmic fraction (gene number 1692),” “intracellular fraction (gene number 2009),” “intracellular fraction (gene number 2013),” and “organelle (gene number 1867).” Molecular functions (MFs) were mainly enriched in “catalytic activity (gene number 908),” “protein binding (gene number 1730),” “cell backbone protein binding (gene number 181),” “nucleotide binding (gene number 360),” and “molecular function (gene number 2324).”

**FIGURE 5:**
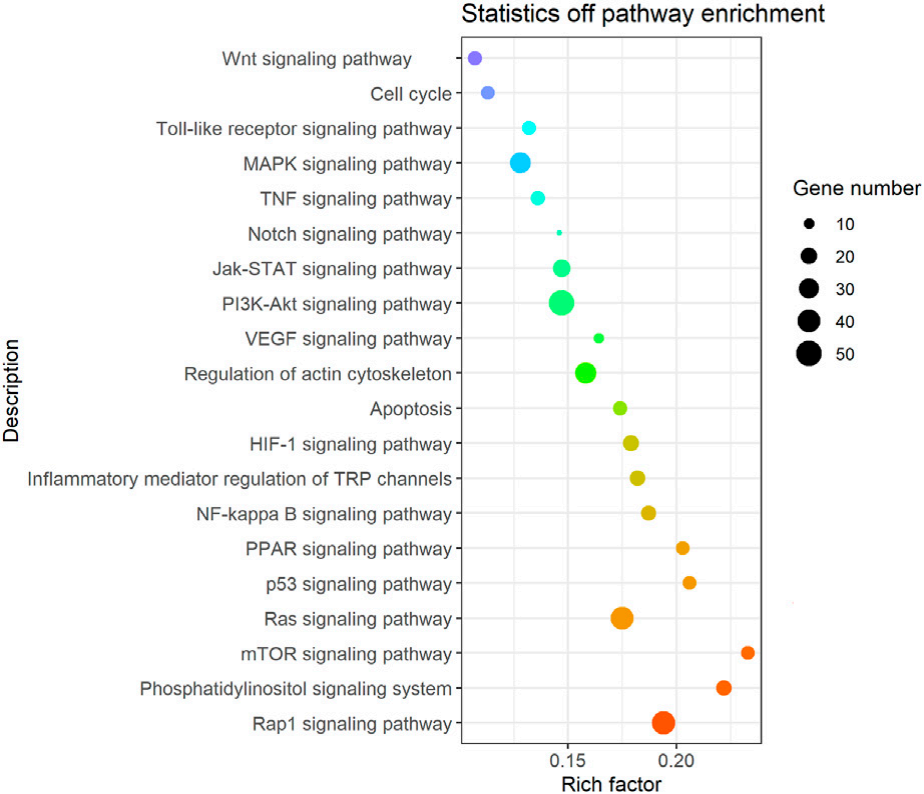
Bubble diagram of the signal pathway associated with the KEGG enrichment of candidate genes. Vertical axes represent pathway name and rich factor. The size of points refers to the number of candidate target genes in the pathway.

It was found that the candidate target genes of these miRNAs with differential expression are rich in 275 relevant metabolic signaling pathways using KEGG enrichment analysis, as shown in Supplementary Table S6 and Figure 5. Such metabolic signaling pathways include inflammatory signaling pathways such as tumor necrosis factor (TNF), nuclear factor-kappa B (NF-kB), and Toll-like receptor signaling pathways (gene numbers 51, 40, and 14, respectively). Fibrotic signaling pathways contain mitogen-activated protein kinase (MAPK), janus tyrosine kinase-signal transducer and activator of transcription (JAK-STAT), rat sarcoma (Ras), Ras-related protein-1 (Rap1), Notch, and wireless/integrated (Wnt) signaling pathways (gene numbers 33, 23, 40, 41, 7, and 15, respectively). Cell cycle, apoptosis, phosphatidylinositide 3-kinase-protein kinase B (PI3K/Akt) signaling pathway, mammalian target of rapamycin (mTOR) signaling pathway, hypoxia-inducible factor-1 (HIF-1) signaling pathway, peroxisome proliferator-activated receptor (PPAR) signaling pathway, and the regulation of the actin cytoskeleton (gene numbers 14, 15, 51, 14, 19, 14, and 34, respectively) are all involved in various crucial BPs. These BPs encompass cell proliferation, differentiation, apoptosis, inflammation, and immune regulation.

### Validation of miRNA expression by qRT-PCR

**FIGURE 6:**
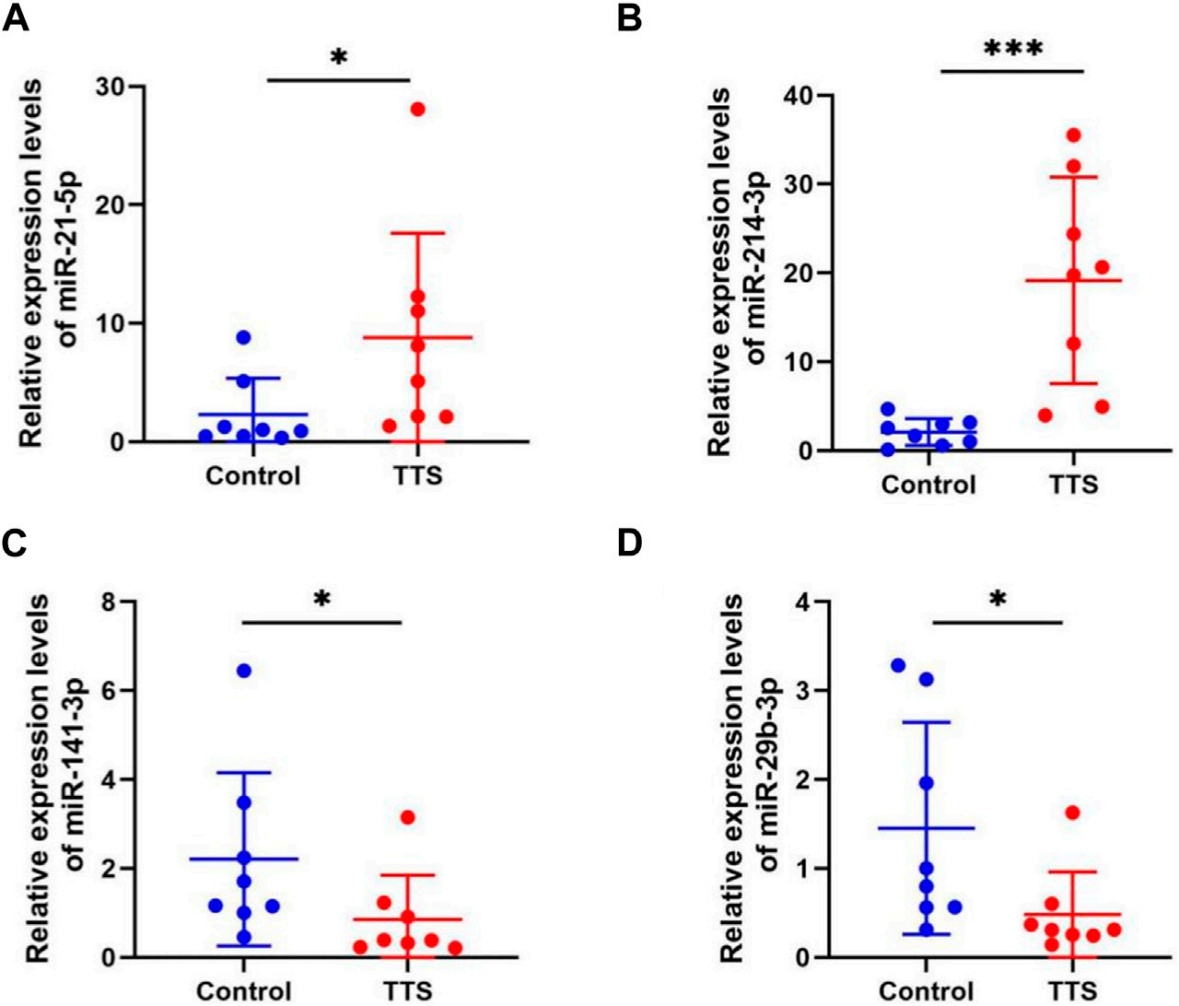
MiRNA expression validation by qRT-PCR in control (n = 8) and TTS groups (n = 8). **(A, B)** MiR-21-5p and miR-214-3p were both significantly upregulated in TTS. **(C, D)** MiR-141-3p and miR-29b-3p were both significantly downregulated in TTS. **Control**: normal bronchial mucosa from pulmonary lobectomy patients. **TTS**: granulation from traumatic tracheal stenosis patients. * *p* < 0.01, *** *p* < 0.001. The 2^-△△CT^ values were used to represent the relative expression of miRNA.

To verify whether the validation of miRNA expression by the analysis of small RNA sequencing data was accurate, four miRNAs (namely, miR-21-5p, miR-214-3p, miR-141-3p, and miR-29b-3p) with differential expression were chosen to detect their differential expression in TTS and normal control by qRT-PCR. Information for the eight TTS patients and the normal control group is presented in Supplementary Table S7. The 2^-△△CT^ values were used to represent the relative expression of miRNA; the △CT and *p* values are shown in Supplementary Table S8. The results showed that the tissues of eight patients in the TTS group reported the significant upregulation of miR-21-5p and miR-214-3p and the significant downregulation of miR-141-3p and miR-29b-3p compared with those in the control one (all *p* values <0.01) (Figure 6).

## Discussion

TTS is primarily characterized by an imbalance between injury and repair due to the activation of various pathogenic factors, such as transforming growth factors, TNFs, interleukin-6 (IL-6), and IL-8 (Karagiannidis et al., 2006; Nicolli et al., 2016). Inflammation, hypoxic injury, abnormal repair, and fibrosis caused by trauma result in the hyperplasia of endotracheal granulation tissues and the formation of fiber scars in TTS (Zhang et al., 2023). MiRNAs are key regulators, which are important to the occurrence and development of many pathological scar hyperplasia and fibrotic diseases and exhibit great potential for disease treatment (Ghafouri-Fard et al., 2021). They have become a new target for the research and development of anti-scar hyperplasia and fibrosis formation, and a research hotspot in cardiopulmonary, liver, and kidney fibrosis diseases (Liu et al., 2019; Wang et al., 2019; Piccolo et al., 2021; Wang et al., 2021; Zhuang et al., 2022).

In the current study, small RNA sequencing was used for detecting the miRNAs of granulation tissues from TTS patients. A differential expression profile of TTS *versus* (vs.) control was successfully constructed. The differential expression profiles of tracheal scar miRNAs were successfully constructed, and 24 miRNAs with significant differential expression were obtained. Among these miRNAs, 13 were upregulated: miR-450a-5p, miR-450b-5p, miR493-3p, miR-93-5p, miR-409-5p, miR-409-3p, miR-21-5p, miR-214-3p, miR-223-5p, miR-379-5p, miR-134-5p, miR-382-5p, and miR-381-3p and 11 were downregulated: miR-29b-3p, miR-30a-3p, miR-195-5p, miR-200a-3p, miR-200a-5p, miR-141-3p, miR-3615, miR-34c-5p, miR-375-3p, miR-449c-5p, and miR-335-5p. QRT-PCR assay demonstrated the upregulation of miR-21-5p and miR-214-3p and the downregulation of miR-141-3p and miR-29b-3p.

Based on the aforementioned results, it was hypothesized that miRNAs are of importance to TTS occurrence and development, which concurs with those seen in other research studies. For instance, inhibiting the expression of miR-1275 has been reported to possibly alleviate airway stenosis (ZHANG et al., 2021). Ye et al. (2021) screened differentially expressed miRNAs in airway scars and normal airway mucosal tissues by gene chips. They successfully verified that miR-127-3p can reduce the secretion of collagens Ⅰ and Ⅲ as well as *α*-smooth muscle actin (α-SMA) and inhibit the proliferation of scar fibroblasts. By consulting existing research workers, it can be found that miR-21-5p was thought to promote the occurrence of the kidney, lung, heart, and other fibrosis in many studies (Wang et al., 2021; Donderski et al., 2022), and miR-214-3p was also strongly related to pulmonary, skin, liver, and kidney fibrosis (Yang et al., 2018; Messner et al., 2021; Yildirim et al., 2021; Xie et al., 2023). On the other hand, it was confirmed that miR-141-3p and miR-29b-3p are protective miRNAs in fibrosis disease (Jin, 2021; Chioccioli et al., 2022; Zhu et al., 2023).

GO and KEGG enrichment analyses demonstrated the acquisition of 2,496 projected target genes for miRNAs with differential expression and their GO enrichment analysis in this research, such as “monoorganism metabolic process,” “monoorganism cell process,” “cell–cell interaction regulation,” and “signal regulation.” Cell components are mainly enriched in the “cytoplasm,” “intracellular fraction,” and “organelle.”. Molecular activities are primarily enriched in “catalytic activity,” “protein,” and “cytoskeletal protein and nucleotide binding.”. According to the aforementioned findings, the target genes of miRNAs are primarily enriched in cells and organelles. A number of intercellular interaction regulation, signal regulation, and other single-organism metabolic and cellular processes were carried out through catalytic activity and molecular functions such as binding proteins, small molecules, and nucleotides.

In this study, 275 related metabolic signaling pathways were examined. Among them, inflammatory signaling pathways such as TNF, NF-κB, and toll-like receptor signaling pathways; fibrosis signaling pathways such as MAPK, JAK-STAT, Ras, Rap1, Notch, and Wnt signaling pathways; and PI3K-Akt, mTOR, HIF-1, and PPAR signaling pathways were found to be enriched. These results indicate the important role of inflammation and fibrosis in TTS development, which is consistent with the studies by Xiao et al. (2020), Fan et al. (2021), and SHI et al. (2021).

However, a few limitations in the present study are supposed to be addressed. The specific mechanisms of miRNAs in TTS should be verified. It is necessary to further explore the role of other target genes *in vivo* and *vitro*. In addition, future studies should focus more on specific molecular mechanisms and regulatory networks. In general, miRNAs may regulate the occurrence and development of TTS. Nevertheless, the mechanism of action and BP is unclear. The deeper action mechanism and the value of these miRNAs are worthy of further investigations.

## Conclusion

In this work, small RNA sequencing was used for detecting the miRNAs of granulation tissues from TTS patients. A differential expression profile of TTS vs control was successfully constructed. In addition, 24 miRNAs with significant differential expression were identified, including 13 upregulated and 11 downregulated ones. Bioinformation technology was adopted to predict 2,496 target genes. These miRNA-target genes were shown to be primarily enriched in cells and organelles with catalytic activity and binding function. QRT-qPCR confirmed the upregulation of miR-21-5p and miR-214-3p and the downregulation of miR-141-3p and miR-29b-3p. These results provide new ideas for further studies and the selection of targets for TTS in the future.

## Data Availability

The datasets presented in this study can be found in online repositories. The names of the repository/repositories and accession number(s) can be found below: BioProject ID PRJNA1046244.
